# A Significant Association of Upper Limb Muscle Strength with Thyroid Function in Overweight and Obese Population: A Study of the Sixth Korea National Health and Nutrition Examination Survey (KNHANES 2014-2015)

**DOI:** 10.1155/2020/7195846

**Published:** 2020-12-02

**Authors:** Jeongmin Lee, Kwanhoon Jo, Jeonghoon Ha, Dong-Jun Lim, Jung Min Lee, Sang-Ah Chang, Moo Il Kang, Min-Hee Kim

**Affiliations:** ^1^Division of Endocrinology and Metabolism, Department of Internal Medicine, Eunpyeong St. Mary's Hospital, College of Medicine, The Catholic University of Korea, Seoul 03312, Republic of Korea; ^2^Division of Endocrinology and Metabolism, Department of Internal Medicine, Incheon St. Mary's Hospital, College of Medicine, The Catholic University of Korea, Incheon 21431, Republic of Korea; ^3^Division of Endocrinology and Metabolism, Department of Internal Medicine, Seoul St. Mary's Hospital, College of Medicine, The Catholic University of Korea, Seoul 06591, Republic of Korea

## Abstract

*Background*. As skeletal muscle is one of main targets of thyroid hormone signalling, an association of thyroid function and muscle strength could be expected. The aim of study is to evaluate the association of free thyroxine (FT4) and thyrotropin (TSH) with upper limb muscle strength, measured by hand grip strength, in subjects with normal FT4 from national representative data. The study utilized the sixth edition of the Korea National Health and Nutrition Examination Survey. After exclusion of subjects with FT4 level out of normal range, a history of thyroid disease or cerebral disease, restricted activity, and incomplete data, a total of 3503 were recruited (age range 19–80 years, 51% male). FT4 positively correlated with upper limb muscle strength (*β* coefficient = −12.84, *p* < 0.001), while TSH did negatively (*β* coefficient = −0.37, *p*=0.002). After adjusting for confounding factors, statistical significance disappeared. However, among subjects with BMI above 23 kg/m^2^, a negative correlation of TSH with upper limb muscle strength was found in a younger age group (19–39 years old) (*β* coefficient = −0.56, *p*=0.021), while FT4 positively correlated with upper limb muscle strength (*β* coefficient = 3.24, *p*=0.019) in an older group (above 40 years old). In overweight and obese subjects, a significant association of thyroid function with upper limb muscle strength was observed in nation-wide representative data. High TSH in a younger group and low FT4 in an older group could be risk factors for decreased upper limb muscle strength in obese population.

## 1. Introduction

Thyroid hormone is essential for normal skeletal muscle development, contractile function, metabolism, and regeneration [1]. Specifically, thyroid hormone regulates normal distribution and changes of fiber types, so-called skeletal plasticity [2–4]. It also exerts important influence on metabolism of skeletal muscle [5, 6] and intracellular calcium regulatory proteins [7]. In addition, thyroid hormone is a fundamental component for myogenesis [8]. Thus, muscular dysfunction could be expected in thyroid dysfunctions. In fact, in both hypothyroidism and hyperthyroidism, muscular symptoms and alterations and dysfunctions of muscle are frequently observed [9–13]. Though several reports failed to present the skeletal muscle abnormalities, an association of subclinical thyroid dysfunctions and muscle dysfunction was also suggested [14, 15]. Even in non-thyroid illness syndrome, a subtle change in thyroid hormone homeostasis represented by a decrease in serum thyroid hormone without subsequent thyrotropin (TSH) increase related acute or chronic illness, alteration in muscle function was suggested in various ways [16]. However, there are only sparse studies [17] which evaluated association serum free thyroxine and TSH with muscle strength in subjects without overt thyroid diseases. Whether subtle changes in thyroid function could cause differences in muscle strength has not been determined yet.

Handgrip strength (HGS) is widely used to assess muscle strength HGS [18]. HGS quantified the maximum static force generated by the hand grip around a dynamometer. It has been used as a reliable marker of overall muscle strength [18, 19]. Muscle strength along with muscle mass and physical performance is a crucial component to define sarcopenia [20]. In addition, muscle strength is associated with physical function, general health, and nutritional status as well as muscle mass [18]. Low HGS, representing weakened muscle strength and low muscle mass, has been suggested to be predictive factor of adverse health outcomes such as physical dysfunction, poor quality of life, and mortality [20]. As HGS possesses importance as a representative of health status and a predictor for health outcomes, evaluation of clinical elements that might lead or link to low HGS could arouse clinicians' interests. In this context, it would be intriguing to evaluate whether thyroid function (specifically, free thyroxine (FT4) and TSH) in subjects without overt thyroid dysfunctions is related to muscle strength or not.

The aim of the study is to evaluate the association of thyroid function and HGS in subjects without overt thyroid diseases from a national representative data.

## 2. Materials and Methods

### 2.1. Study Population and Data Collection

This study was a retrospective study based on data from the sixth edition of the Korea National Health and Nutrition Examination Survey (KNHANES) conducted between 2014 and 2015. The survey has been performed by trained interviewers on an annual basis for monitoring the health and nutritional status of Koreans. This study complied with the ethical standards of the Helsinki Declaration and was approved by the Catholic University of Korea, Catholic Medical Center, Eunpyeong St, Mary's Hospital Institutional Review Board (IRB approval No. PC19ZISI0154). Written informed consent was exempted due to retrospective review. Initially, there were 22948 adults recruited from KNHANES VI. Among them, individuals were excluded based on the following criteria: age less than 19 years, FT4 level out of normal range (0.89–1.76 ng/dL), a reported history of thyroid disease, restricted activity, history of cerebral disease, and unavailable data for upper limb muscle strength, and urine iodine. After exclusion, a total of 3503 subjects were finally included in this analysis. Among the 3503 subjects, 51.0% were men (*n* = 1788). The characteristics and HGS measurements of the study subjects are summarized in Table 1. The mean age was 43.8 years (range 19–80 years), 2027 subjects were over age 40 (57.9%), the mean BMI was 23.88 kg/m^2^ (range 14.65–43.82), the right hand was the most dominant (88.6%), and the mean HGS was 33.39 ± 0.31 kg.

### 2.2. Demographic Characteristics and Survey on Status of Health and Nutrition

Demographic data and medical histories were obtained from self-reported questionnaires and personal interviews by trained medical staff. Age, body weight, height, smoking and drinking habits, exercise status, and presence of diabetes were collected. Body mass index (BMI) was calculated as weight divided by height squared (kg/m^2^). Smoking status was divided into current smokers and non-smokers including ex-smokers. Drinking status was classified as non-to-moderate drinking and heavy drinking (more than 30 g/day of alcohol). Regular exercise was defined as moderate physical activity for at least 2 hours and a half or severe intensity exercise more than an hour and 15 minutes or at least 2 hours and a half for moderate-to-severe intensity (severe exercise 1 min = moderate exercise 2 min). Presence of diabetes was defined as history of diagnosis or taking antidiabetic medication.

### 2.3. Muscle Function Measurement-HGS

Upper limb muscle strength was measured by handgrip strength because its functional importance in daily activities is well established. HGS was measured using a digital hand dynamometer which measures 5.0–100.0 kg of force (Digital Grip Dynamometer, T. K. K. 540, 1 Takei Scientific Instruments Co., Ltd., Tokyo, Japan). The method for handgrip analysis used in this study has been validated and extensively used in clinical trials [21–23]. Grip strength was measured while subjects were in a standing position. Starting with the dominant hand and alternating, each hand was tested three times. The average HGS value of the dominant hand was used as the final HGS [24] according to consensus from Asian Working Group of Sarcopenia [19]. In the case of ambidextrous subjects, the higher HGS average between the right and left hands was used. A previous analysis of the reference ranges of HGS in the Korean population [24] revealed a positive correlation of BMI with HGS, and peak HGS was reached around the age of 35–39 years. In subgroup analysis, the population was stratified into groups of obese (including overweight) or non-obese and young (19–39 years) or old (≥40 years).

### 2.4. Measurement of Thyroid Function

All blood samples were collected year-round in the morning after 8 hours of fasting. Blood samples from all subjects were immediately processed, centrifuged, liquated, and sent to the Central Testing Institute in Seoul, Korea, for analysis within 24 hours. FT4 was measured with using an electrochemiluminescence immunoassay, E-TSH kit (Roche Diagnostics, Manheim, Germany), and TSH was measured with E-TSH kit (Roche Diagnostics, Manheim, Germany). The reference for FT4 was 0.81–1.76 ng/mL and that for TSH was 0.35–5.50 mIU/L.

### 2.5. Statistical Analysis

To provide nationally representative prevalence estimates, statistical procedures were performed to reflect the complex sampling design and sampling weights of the KNHANES VI. The SAS® PROC SURVEY module was used to consider strata, clusters, and weights. Based on the characteristics of the data, the results were expressed as the means ± standard error (SE), geometric means (95% confidence interval [CI]), or percentages, as appropriate. Characteristics of each gender were compared with the chi-square test for dichotomous variables and independent *t*-tests for continuous variables. Multivariate logistic regression analysis for sex, smoking and drinking status, diabetes, and exercise habits was performed. To determine clinical significance from the analysis, the population was categorized into groups: normal and overweight or obesity (BMI >23 kg/m^2^), and an age range of 19–39 or >40 years. All statistical analyses were performed using SAS® software, version 9.4. (SAS Institute Inc., Cary, NC, USA). A *p*-value <0.05 was considered statistically significant.

## 3. Results

**Figure 1:**
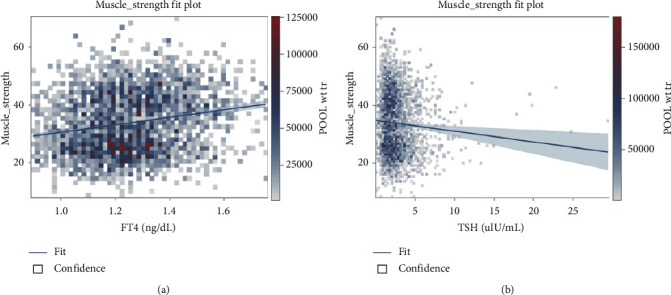
Univariate linear regression analyses of factors associated with muscle strength. In univariate analysis, free thyroxine (FT4) positively correlated with upper limb muscle strength (*β* coefficient = −12.84, *p* < 0.001), while thyrotropin (TSH) did negatively (*β* coefficient = −0.37, *p*=0.002).

In the linear regression with univariate analysis, FT4 positively correlated with upper limb muscle strength (*β* coefficient = −12.84, *p* < 0.001), while TSH demonstrated a significantly negative correlation (*β* coefficient = −0.37, *p*=0.002) (Figure 1). After adjustment for age, sex, smoking and drinking habits, diabetes, and exercise, there was no significant correlation between HGS and FT4 or TSH (Table 2).

In the analysis of subjects with BMI <23 kg/m^2^, there was no significant association between HGS and thyroid function in either age group. However, in the overweight or obese group (BMI ≥23 kg/m^2^), there was a significant negative correlation between TSH and HGS in the 19–39-year-old group (*β* coefficient = −0.56, *p*=0.0209), and there was a significant positive correlation between FT4 and HGS in the >40-year-old group (*β* coefficient = -3.24, *p*=0.0002) (Table 3). Additional analyses according to quartiles of FT4 and TSH were performed in subjects with BMI ≥23 kg/m^2^. HGS significantly decreased as quartiles of FT4 increased in the younger age group (*p*=0.0249). On the other hand, HGS increased as TSH quartiles decreased in subjects aged >40 years, but the difference was not statistically significant (*p*=0.0513) (Table 4).

## 4. Discussion

The purpose of this study was to elucidate the relationship of FT4 and TSH with upper limb muscle strength based on the nationwide representative data collected in the 2014–2015 KNHANES VI. The subjects included in this study had serum FT4 levels within normal range; thus, the study population was composed of subjects with normal or relatively subtle changes in thyroid function. Many previous studies have demonstrated a strong relationship between BMI and HGS [23, 25, 26]; thus, further subgroup analysis was performed according to BMI. In addition, considering that a positive correlation between age and HGS at 19–39 years of age and a negative correlation over age 40 have been reported [27], subjects were categorized into two groups based on age (19–39 years and >40 years). Though no significant association was found in the non-obese (underweight and normal BMI) population, a negative correlation of TSH with HGS in younger subjects and a positive correlation of FT4 with HGS in the older subjects were found in the overweight and obese group.

As skeletal muscle is one of the main targets of thyroid hormones, it has been postulated that an alteration in thyroid function would have a significant impact on skeletal muscle physiology and structure. Thyroid hormones are essential for normal skeletal fiber differentiation by transcription of myogenic regulatory factors [28]. They also play a crucial role in the differentiation of muscle fiber types during postnatal development as evidenced by abnormal skeletal muscle fiber expression in the absence of thyroid hormone [3, 4]. Skeletal muscle plasticity, ability to changing muscle fiber types after development, depends on thyroid hormone signalling [2–4]. Thyroid hormones determine muscle energy expenditure by regulating several proteins involved in energy metabolism such as retinol-binding-protein-1, glycogen synthase 1, muscle pyruvate kinase [29, 30], myosin, and sarcoplasmic/endoplasmic reticulum Ca2+-ATPase (SERCA) [3, 7]. They also control other aspects which are important to energy metabolism, such as mitochondrial content and activity in skeletal muscle [5] as well as glucose transporter [6]. Thyroid hormone is involved in myogenesis by virtue of regulating skeletal muscle stem cells [8]. The detailed roles of thyroid hormones in skeletal muscular physiology are well described in the literatures [1, 31]. In addition to the roles of thyroid hormones in skeletal muscle, TSH is also suggested to have a role in muscular metabolism. Though the exact role of TSH in skeletal muscle physiology has not been fully evaluated, the presence of TSH receptors and a functional role of TSH in energy metabolism in skeletal muscle have been presented [32, 33]. Therefore, correlation of muscle strength and thyroid function, which would be represented by thyroid hormone and TSH, could be suspected.

Clinical findings in patients with overt hyper- and hypothyroidism, such as alterations in deep tendon reflex, have suggested an association between thyroid function and skeletal muscle function. There are several studies which have suggested the association between thyroid dysfunction and muscle strength [11–13, 22, 34]. In both overt hypo- and hyperthyroidism, myopathy which results in muscle weakness, aching, and cramping is frequently observed, and those symptoms can be relieved in response to hormone replacement [19, 22]. Olson et al. reported decreased muscle mass and strength in patients with overt hypothyroidism and recovery of myopathy after treatment [12]. Further, additional musculoskeletal manifestations such as adhesive capsulitis, carpal tunnel syndrome, and fibromyalgia syndrome were also frequently detected in patients with thyroid dysfunctions [9]. In subclinical thyroid dysfunctions, no consistent results were found in terms of association with muscular dysfunction. While no clear association of subclinical thyroid dysfunction and muscle strength was observed in some studies [14, 35, 36], other studies reported reduced muscle strength related to subclinical thyroid dysfunctions [13, 15]. Compared to subjects with euthyroidism, the strength of the flexor and extensor muscles was reduced in subjects with hyperthyroidism, and muscle function improved after the restoration of normal thyroid levels even in elderly patients with subclinical hyperthyroidism [37].

The measurement of HGS, which is commonly used to estimate muscle strength, has clinical implications because it is associated with not only health and nutritional status but also various health outcomes [18, 20]. There were significant correlations shown between HGS and gender [38, 39], hand length or forearm circumference [40, 41], various chronic diseases [42], age, and BMI in previous studies. The age at which HGS shows peak is generally around the age of 40 years [45–47], and how HGS changes as a person ages shows very similar patterns among different ethnicities [46, 48]. Positive correlations of HGS with BMI were also observed in various ethnicities [40, 49, 50]. However, most of the parameters, which were known to be correlated with HGS, were anthropometrics.

In this study, we found a significant association of thyroid function with upper limb muscle strength in subjects with BMI ≥23. In this population, different patterns of association were noticed according to age. Specifically, TSH showed a negative correlation with HGS in a younger group, while FT4 showed a positive correlation in an older group. Firstly, a plausible explanation for why the significant association between thyroid function and HGS was found only in overweight or obese population could be made based on cross-talk between thyroid and adipose tissue [51]. In fact, obesity itself has impact on muscle strength and structure [52] and, as shown by the impaired muscle regeneration in obese and diabetic mice [53], the regeneration capability of skeletal muscle would be impaired compared to that in non-obese subjects impaired skeletal muscle regeneration capacity in obese individuals [25]. It has been suggested that skeletal muscle deterioration related to obesity is mediated by the secretion of cytokines by adipose tissue [54] and/or fat infiltration into muscles which induces skeletal lipotoxicity and insulin resistance [55]. Thus, considering the cross-talk between thyroid and adipose tissue, subtle changes in thyroid function in obese subject would potentiate the impact of obesity on muscle. Secondly, the association of thyroid hormone levels and upper limb muscle strength in relation to age, namely, the negative correlation of TSH in younger subjects and the positive correlation of FT4 in older subjects, could be explained mainly by the changes in skeletal muscle during the aging process. As one ages, the changing proportion of muscle fiber types causes alterations in skeletal muscle structure and metabolism [56–58]. The age-related changes of decreased muscle mass and number of fibers were associated with the distribution of muscle fiber denervation leading to the loss of motor units [57]. It could be assumed that changes of aging muscle, in terms of mass and component, would result in different responsiveness to thyroid hormone or TSH. In addition, during aging, while FT4 levels remain stable as a person ages, serum TSH increase is prominent [17] and muscle strength also decreases with age [24, 27]. Thus, the impact of TSH on muscle strength might be attenuated in older subjects.

An advantage of this study is that it is based on data that is representative of the population. In addition, this might be the first study which demonstrates a correlation between HGS and thyroid function according to age and BMI. However, there were several limitations. First, serum T3, which is a crucial regulator of skeletal muscle metabolism and homeostasis, was not measured in the KNHANES VI. Therefore, we could not have investigated the relationship between bioactive thyroid hormone and upper limb muscle strength. Second, although we adjusted for possible confounding factors, we could not adjust for lean body mass or fat-free mass per se. Thus, the correlation between muscle strength and adiposity could have causal interferences. Third, because this study was a cross-sectional study, the complex mechanism between thyroid dysfunction and upper limb muscle strength could not be clarified. Moreover, due to analysing the collected data and using only measurement of upper limb strength, we could not have confirmed the correlation between HGS and overall muscle strength and physical performance, which may be crucial in evaluating muscle function that was not evaluated. Fourth, the subjects were categorized into the two groups (19–39 years and over 40 years) in this study: the older group included young, middle-aged, and old group untypically. However, the average grip strength for age and gender was reported to be in a varying range [59, 60]. Therefore, the age groups were classified according to the Korean reference [24].

## 5. Conclusions

This study demonstrated a significant association between thyroid function and upper limb muscle strength in overweight and obese subjects. Interestingly, different association patterns were observed between the younger and older age groups. The impacts of thyroid function on upper limb muscle strength might differ between the non-obese and obese population and different mechanisms linking thyroid and muscle function might exist according to age.

## Figures and Tables

**Table 1:** Baseline characteristics.

Characteristics	Unweighted sample size (*n* = 3503)	Weighted sample size (*n* = 10430802)
Sex (men, %)	1788 (51.0)	5315537 (51.0%)
Age, years	43.8 ± 14.9 (19–80)	43.7 ± 0.3 (19–80)
≥40 (*n*, %)	2027 (57.9)	(57.2)
<40 (*n*, %)	1,476 (42.1)	(42.8)
BMI, kg/m^2^	23.88 ± 3.60 (14.65–43.82)	23.84 ± 0.07 (14.65–43.82)
≥23	1981 (56.6)	
<23	1522 (43.5)	
Dominant hand	3430	
Right (*n*, %)	3,040 (88.6)	(88.8)
Left (*n*, %)	182 (5.3)	(5.2)
Both (*n*, %)	208 (6.1)	(6.0)
Handgrip strength, kg	33.39 ± 10.31 (8.08–70.57)	35.07 ± 0.20 (8.08–70.57)
FT4, ng/dL	1.25 ± 0.16 (0.89–1.76)	1.25 ± 0.00
TSH, uIU/mL	2.61 ± 1.86 (0.01–29.50)	2.59 ± 0.03

Values are presented as number (%) or mean ± SD (range). BMI, body mass index (calculated as weight divided by height squared (kg/m^2^)). FT4, free thyroxine. TSH, thyrotropin.

**Table 2:** Multivariate regression analyses of factors associated with upper limb muscle strength (weighted).

	FT4 *β* (95% CI)	*P-*value	TSH *β* (95% CI)	*p-*value	LOG_TSH *β* (95% CI)	*p-*value
Model 1	12.84 (10.42,15.26)	<0.0001	−0.37 (−0.61, −0.14))	0.0018	−0.95 (−1.58, −0.33)	0.0030
Model 2	0.02 (−1.56, 1.60)	0.9791	−0.03 (−0.17, 0.10)	0.6297	−0.05 (−0.44, 0.35)	0.8087
Model 3	0.31 (−1.31, 1.93)	0.7108	−0.02 (−0.16, 0.12)	0.7864	−0.05 (−0.47, 0.37)	0.8103
Model 4	0.27 (−1.35, 1.89)	0.7450	−0.01 (−0.15, 0.13)	0.9054	−0.02 (−0.44, 0.40)	0.9241

Model 1: crude. Model 2: adjustment for age, sex, BMI. Model 3: model 2 + smoking, drinking, diabetes. Model 4: model 3 + exercise. CI, confidential interval. FT4, free thyroxine. TSH, thyrotropin.

**Table 3:** Relation between upper limb muscle strength with thyroid hormone status according to BMI and age.

BMI and age group	FT4 *β* (95% CI)	*P-*value	TSH *β* (95% CI)	*P-*value
BMI <23 (*n* = 1522)	0.54 (*−*181, 2.89)	0.6515	0.04 (−0.13, 0.21)	0.6450
Age 19–39 (*n* = 780)	*−*0.28 (*−*3.78, 2.93)	0.8658	−0.04 (−0.29, 0.20)	0.7264
Age ≥40 (*n* = 742)	2.52 (*−*0.82, 5.85)	0.1383	0.10 (−0.12, 0.32)	0.3862
BMI ≥23 (*n* = 1981)	0.22 (*−*0.205, 2.49)	0.8494	−0.03 (−0.22, 0.16)	0.7682
Age 19–39 (*n* = 696)	*−*2.90 (*−*7.11, 1.30)	0.1750	−0.56 (−1.04, −0.09)	0.0209
Age ≥40 (*n* = 1285)	3.24 (0.53, 5.95)	0.0192	0.17 (−0.08, 0.41)	0.1836

Corrected by sex, smoking, drinking, diabetes, and exercise. BMI, body mass index (calculated as weight divided by height squared (kg/m^2^)). CI, confidential interval. FT4, free thyroxine. TSH, thyrotropin.

**Table 4:** Relation of upper limb muscle strength with quartiles of TSH and FT4 in overweight and obese subjects (BM ≥23 kg/m^2^).

Age group	TSH				*P-*value	FT4				*P*-value
	Q1	Q2	Q3	Q4		Q1	Q2	Q3	Q4	
Age 19–39 (*n* = 696)	40.88	39.25	39.82	38.41	0.0249	36.42	39.49	38.73	41.86	0.3236
Age ≥40 (*n* = 1285)	36.43	36.82	36.09	33.87	0.1533	33.32	35.62	36.61	38.74	0.0513

Corrected by sex, smoking, drinking, diabetes, and exercise. BMI, body mass index (calculated as weight divided by height squared (kg/m^2^)). FT4, free thyroxine. TSH, thyrotropin. TSH (mIU/L), Q1 ≤1.51, 1.51< Q2 ≤2.21, 2.21< Q3 ≤3.17, 3.17 Q4 ≤29.50. FT4 (ng/dL), Q1 ≤1.13, 1.13Q2 ≤1.23, 1.23 < Q3 ≤1.35, 1.35 < Q4 ≤1.76.

## Data Availability

The datasets used and analysed during the current study could be made available upon reasonable request to the corresponding author.
